# Word superiority and sentence superiority effects in post-cued letter-in-string identification

**DOI:** 10.3758/s13414-025-03059-w

**Published:** 2025-04-08

**Authors:** Stéphanie Massol, Jonathan Grainger

**Affiliations:** 1 Laboratoire d’Étude des Mécanismes Cognitifs (EMC), Institut de Psychologie, Université Lumière Lyon 2, 5 Avenue Pierre Mendès France, 69676 Bron cedex, France; 2 Centre de Recherche en Psychologie et Neurosciences, CNRS & Aix-Marseille, Marseille, France; 3 Institute of Language, Communication and the Brain, Aix-Marseille University, Marseille, France

**Keywords:** Sentence superiority, Word superiority, Letter-in-string identification

## Abstract

We examined word superiority and sentence superiority effects in post-cued letter identification by embedding target letters in a letter string that was part of a sequence of letter strings separated by spaces. Experiment 1 compared letter identification in words versus random consonant strings (i.e., nonwords), thus involving three conditions: grammatical word (e.g., HE RUNS OVER THERE; the target being the letter V), ungrammatical word (e.g., THERE HE OVER RUNS), and nonwords (e.g., THPRN HJ GVTR LPDKS). Stimuli were displayed for 500 ms and post-masked. Letter-in-word identification was greater in the grammatical than in the ungrammatical word context (a sentence superiority effect, SSE). Moreover, letter-identification accuracy was greater in words than in nonwords (a word superiority effect, WSE). Experiment 2 used pronounceable pseudowords instead of nonwords and replicated the SSE and WSE seen in Experiment 1, with the size of the WSE being substantially reduced relative to Experiment 1. Experiment 3 tested letter identification in words, pseudowords, and nonwords, either in a grammatical or in an ungrammatical context. We again found a significant SSE on letter-identification accuracy combined with the standard pattern of the WSE (word > pseudoword > nonword). Finally, the classic WSE pattern was also found when stimuli were presented in isolation in Experiment 4.

## Introduction

The word superiority effect (WSE) is one of the key phenomena in motivating theoretical developments aimed at understanding the basic mechanisms involved in how skilled readers process written words. First reported by Cattell (1886), the phenomenon was boosted by the methodological innovations brought by the work of Reicher (1969) and Wheeler (1970). Reicher and Wheeler used a post-cued two-alternative forced-choice (2 AFC) task in order to rule out: (1) memory accounts of the WSE (i.e., it is easier to remember a word than a nonword); and (2) sophisticated guessing accounts of the phenomenon whereby a participant could guess the identity of a letter given partial information about the word that was presented. In the present study we opted for a simpler post-cued letter-identification task that mimics the task typically used to study the sentence superiority effect, as described below.^1^ Independently of the task used to study the WSE, prior research (e.g., Adams, 1979; Grainger et al., 2003; McClelland, 1976; Rumelhart & McClelland, 1982) has systematically found that letter identification is more accurate when the letter is embedded in a word compared with a nonword, and, furthermore, that letter identification is better in a pronounceable nonword (henceforth referred to as pseudowords) compared with unpronounceable nonwords (typically random consonant strings). The present study investigated the impact of the extended surrounding context on the WSE. That is, we tested letter identification with target letters embedded in words, pseudowords, or nonwords, which were themselves embedded in sequences of words (that could be grammatically correct or not) or sequences of nonwords (see Table 1).

**Table 1:** Examples of the different conditions tested in Experiments 1–3 provided in English for convenience

Experiment 1	
Grammatical word:	HE LOOKS OVER THERE
Ungrammatical word:	THERE HE OVER LOOKS
Nonword:	THPRN HJ GVTR LPDKS
Experiment 2	
Grammatical word:	HE LOOKS OVER THERE
Ungrammatical word:	THERE HE OVER LOOKS
Pseudoword:	THORU HO AVIR LEIKS
Experiment 3	
Grammatical word:	HE LOOKS OVER THERE
Ungrammatical word:	THERE HE OVER LOOKS
Grammatical pseudoword:	HE LOOKS AVIR THERE
Ungrammatical pseudoword:	THERE HE AVIR LOOKS
Grammatical nonword:	HE LOOKS GVTR THERE
Ungrammatical nonword:	THERE HE GVTR LOOKS

*Note*. Examples are given in English for convenience. In these examples the target is the letter “V” (underlined here but not in the Experiment). Target letters could be embedded in any of the four word/nonword locations, but never occupied an external position (i.e., first or last) in the word/nonword

Before presenting the specific aims of the present study, we first note that Scaltritti et al. (2021) had previously compared identification of a single word at a post-cued location in ungrammatical sequences of three unrelated words (e.g., dog pin lot) with identification of a single letter at a post-cued location in a sequence of three nonwords (e.g., deg ltr fnt). In this example (in English for convenience), identification of “dog” at the first position in the three-word sequence was compared with identification of the letter “d” at the first position in the nonword sequence as well as identification of the letters “o” and “g” at the second and third positions of different nonwords located at the first position in the sequence of nonwords. The aim of that study was to examine the extent to which word-in-sequence identification is driven by the ease of identification of the word’s component letters. The results of that study suggested that that was indeed the case. This was taken as support for a model of reading (in written languages that use an alphabetic script) according to which parallel letter processing subsequently enables parallel word processing. That is, support for the parallel, cascaded nature of letter and word processing during reading.

The overall aim of the present study was to provide a further test of a parallel, cascaded, interactive account of reading (e.g., Rumelhart, 1977). This theoretical approach was first put to test by McClelland and Rumelhart (1981) with a focus on the letter-word interface. The three basic principles of this approach are: (1) parallel processing (within the limits imposed by visual acuity, spatial attention, and crowding; see, e.g., Grainger et al., 2016); (2) cascaded processing – that is, processing at a higher level begins before processing of the lower level is complete; and (3) interactive processing – that is, as soon as processing at the higher level begins it feeds back information to the lower level. A further constraint, illustrated in the work of Brossette et al. (2022), is that principles 2 and 3 only apply to adjacent levels of processing (i.e., letters-words; words-sentences). In the present work we focus on the implications of principles 2 and 3 for a general theory of reading.

The cascaded interactive-activation approach was initially applied to the letter-word interface in order to explain the WSE (McClelland & Rumelhart, 1981). According to this account of the WSE, activated letter representations subsequently activate all compatible word representations, which then feed back information to on-going letter processing, hence facilitating identification of letters that are parts of a word. A test of this general theory of reading was subsequently extended to the word-sentence interface in the work of Snell and Grainger (2017). Snell and Grainger reported a sentence superiority effect (SSE) using a post-cued partial report procedure, and as such can be considered to be the word-in-sentence equivalent of the letter-in-word WSE (see Grainger, 2024). In Snell and Grainger (2017), a sequence of words was briefly presented simultaneously and followed by a backward mask accompanied by a post-cue indicating which word in the sequence is to be reported. The post-cue in SSE experiments indicates the position of one word in a sequence of words that can either be grammatically correct (e.g., the man *can* run) or an ungrammatical re-ordering of the same words (e.g., man run *can* the). The target word (italicized here for convenience) is located at the same position in the grammatical and ungrammatical sequences. Accuracy of post-cued report is greater when the target word is embedded in a grammatically correct sequence compared with ungrammatical sequences (Massol et al., 2021; Snell & Grainger, 2017; Wen et al., 2019). In the same way that the WSE has been taken to reflect cascaded-interactive processing at the letter-word interface (e.g., McClelland & Rumelhart, 1981), in the above-cited papers the SSE has been taken to reflect cascaded-interactive processing at the word-sentence interface (for reviews, see Grainger, 2018, 2024).

The similar procedures that are used (i.e., post-cued partial report) and the similar conclusions that are drawn with respect to the WSE and the SSE led us to hypothesize that the two phenomena should have cumulative effects when examined in the same experiment. That is, a grammatical context should enhance the WSE compared with an ungrammatical context. This would arise by the grammatical context providing feedback to word-level processing that in turn would generate improved letter-identification accuracy via word-letter feedback. In other words, we expected to observe a combination of an SSE and a WSE that would reflect cumulative effects of cascaded-interactive processing across letter, word, and sentence levels. Experiments 1–3 put this hypothesis to test with different types of nonword stimuli and different types of global (sentential) context. Finally, Experiment 4 tested the same word, pseudoword, and nonword stimuli as in Experiment 3, but in the absence of any global context (i.e., isolated presentation of words, pseudowords, and nonwords).

## Experiment 1

### Methods

#### Participants

A total of 90 participants (52 male) with a mean age of 31.4 years (SD = 9.34, min–max: 18–60 years) were recruited via the Prolific platform (Palan & Schitter, 2018). All participants were native speakers of French, with no history of neurological impairment or developmental disorders. Prior to the beginning of the experiment, participants were informed that data would be collected anonymously, and they provided informed consent before the experiment was initiated.

#### Materials

We first constructed 240 grammatically correct sentences that consisted of four French words. Word length was from three to five letters, and the summed frequency of the content words was 5.18 Zipf; SD = 0.66 (log10 occurrences per million words + 3; van Heuven et al., 2014). For the purpose of the letter-identification task, one letter in every sentence was marked as the critical target. This target letter was always a consonant and appeared in an internal position within the word (e.g., “elle vend son pain”, with the letter “n” as letter target (the English translation is “she sells her bread,” but as in this example, direct translations do not always respect the design of the experiment, such as having a double letter “l” in “sells.” Therefore, we provide English examples that are not translations of the French materials in Table 1). The target letter was equally distributed among the four words in sequences. Therefore, there were 60 critical target letters for each word position. We then constructed grammatically incorrect word sequences based on this set of 240 sentences by scrambling word order in the correct sentences but keeping the position of the word in which the target letter was marked in the same position in the sequence. Moreover, we made sure that the target letter appeared at exactly the same position in both conditions (e.g., the ungrammatical version of the grammatical sequence described above was: “pain vend elle son” such that the target letter “n” appeared in the same position in the same word at the same location in the sequence). Finally, we created 240 sequences of nonwords, derived from the ungrammatical word sequences. To do so, for each ungrammatical sequence (e.g., “pain vend elle son”), each vowel was replaced with a consonant that was not present within the word (e.g., “plsn vbnd fllk sdn”). Again, the target letter (“n” in “vbnd” in this example) was always at the same position in the nonword, and the nonword was at the same position in the sequence of strings compared with the “grammatical word” and “ungrammatical word” conditions. These three sets of 240 sequences were separated into three subsets to create three lists of experimental stimuli presented to different participants. In this way, participants saw each sequence only once but were tested in all conditions with different sequences. Across participants, each set of four-word sequences occurred only once in its grammatically correct version (e.g., “elle vend son pain”), in the ungrammatical version (e.g., “pain vend elle son”), or in the nonword version (e.g., “plsn vbnd fllk sdn”: see Table 1 for a summary of the conditions).

#### Procedure

**Fig. 1:**
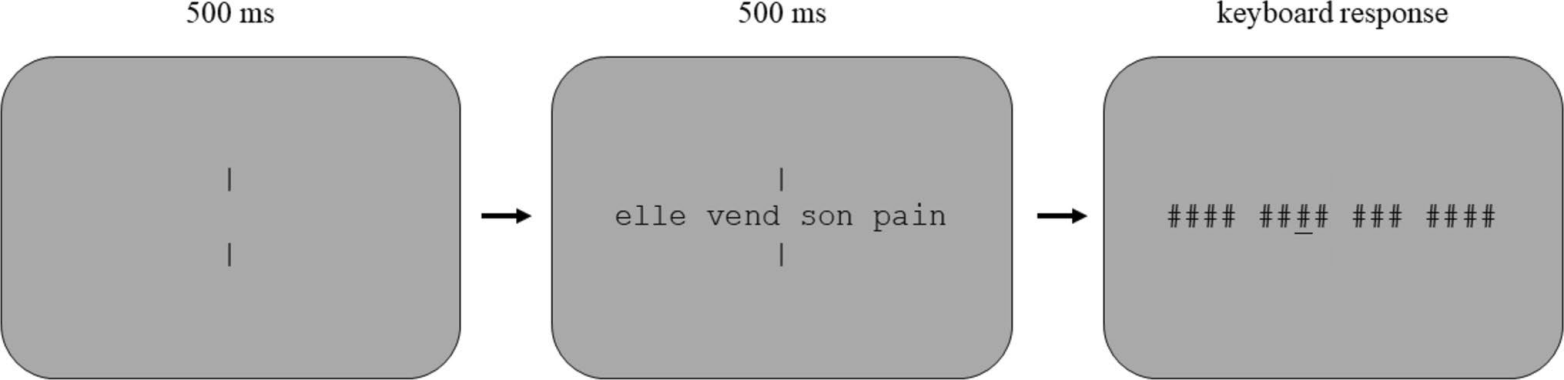
The post-cued partial report, rapid parallel visual presentation (RPVP) procedure used in Experiments 1–3. Participants had to type in the letter they thought had been presented at the post-cued location indicated by the underlined hash mark (#)

Participants performed this task on-line. Stimuli were presented using Labvanced software (Finger et al., 2017) on the personal computer screen of the participant. All sequences were presented as black letters in lowercase (font size 28 pt) and centered vertically and horizontally on a light gray background. Written instructions were given at the beginning of the experiment. Participants were informed that a sequence of four-word strings was going to be displayed briefly, followed by a pattern mask. They were asked to decide which letter they had seen in the position indicated by an underline cue during the presentation of the backward mask (i.e., a post-cue). Each trial began with the presentation of two vertical bars positioned at the center of the screen that remained on the screen until the stimulus sequence was replaced by a backward mask. Participants were asked to focus their attention between the two vertical bars (i.e., the center of the screen) at the beginning of each trial. After 500 ms, the stimulus sequence was displayed centered on the screen for 500 ms. This was then followed by a backward mask composed of hash marks at all positions that were occupied by a letter in the previous string and accompanied by an underlined cue to indicate the target letter location. Participants could type their response at this point, and their response appeared in a box located slightly below the string of hash marks (see Fig. 1). There was no time pressure to respond, hence our dependent variable was accuracy. Once the return key was pressed, the next trial was displayed. A practice session (12 trials) was administered before the main experiment to familiarize participants with the procedure. The 240 trials of the main experiment were presented in a random order. The experiment lasted approximately 15 min.

#### Statistical power

With 90 participants and 80 items per condition the power of the experiment was largely superior to that recommended by Brysbaert and Stevens (2018).

### Results and discussion

First, the datasets from four participants were excluded, based on an overall error rate above 40%. We used generalized (logistic) linear mixed-effects models (LMEs) to analyze our data, with items and participants as crossed random effects and including by-item and by-participant random intercepts and random slopes (Baayen et al., 2008; Barr et al., 2013). The logistic mixed-effects model included Grammaticality (grammatical word, ungrammatical word, vs. nonword) as fixed factor. The models were fitted with the glmer function from the lme4 package (Bates et al., 2014) in the R statistical computing environment (version 4.0.220, R Core Team, 2022). The maximal random effects structure that converged was one including by-participant and by-item random intercepts as well as by-participant random slopes for the Grammaticality factor. The following analyses were conducted taking the ungrammatical word condition as reference. We report regression coefficients (*b*), standard errors (*SE*) and *z*-values for all factors. Fixed effects were deemed reliable if |*z*| > 1.96. Mean accuracy rates for each of the experimental conditions are presented in Table 2.

**Table 2:** Mean accuracy (% correct) in the grammatical word condition, the ungrammatical word condition, and the nonword condition in Experiment 1

Grammatical word	Ungrammatical word	Nonword
91.80 (5.79)	85.33 (7.47)	19.85 (8.43)

*Note*. Within-participant 95% confidence intervals are shown in parentheses

Letter identification accuracy was higher in the grammatical word condition than in the ungrammatical word condition (91.80% vs. 85.33%; *b* = 0.83, *SE* = 0.06, *z* = 11.98, *p* <.001). Accuracy was also higher in the ungrammatical word condition than in the nonword condition (85.33% vs. 19.85%; *b* = − 3.73, *SE* = 0.09,* z* = − 38.52, *p* <.001).

In sum, we observed an SSE on letter-in-string identification accuracy as well as replicating the classic WSE. That is, letter-identification accuracy was greater when letters were embedded in a word that was part of a grammatically correct sequence of words, and letter-identification accuracy was greater in words compared with nonwords. Experiment 2 aimed to extend these findings by replacing the nonwords of Experiment 1 with pronounceable pseudowords. This was important given that prior research has found a robust WSE when comparing letter-in-word identification with letter-in-pseudoword identification, that was albeit much smaller in magnitude than the effect observed when comparing words and nonwords (Adams, 1979; Grainger et al., 2003; McClelland, 1976; Rumelhart & McClelland, 1982). In Experiment 2 we first examined the impact of a global context on the WSE comparing words with pseudowords in contexts that matched the target string (i.e., words for word targets and pseudowords for pseudoword targets). This therefore provided a more stringent test of the effect of global context on the WSE compared with Experiment 1 (word vs. nonword), with the embedding target string here being either a word or a pseudoword.

## Experiment 2

### Methods

#### Participants

A total of 90 participants (55 male) with a mean age of 33 years (SD = 10.77, min–max: 20–65 years) were recruited via the Prolific platform (Palan & Schitter, 2018). All participants were native speakers of French, with no history of neurological impairment or developmental disorders. Prior to the beginning of the experiment, participants were informed that data would be collected anonymously, and they provided informed consent before the experiment was initiated.

#### Materials

The exact same materials used in the grammatically correct sentences and ungrammatical word sequences tested in Experiment 1 were used in Experiment 2. Here, we replaced the sequences of nonwords with sequences of pseudowords. To do so, for each ungrammatical word sequence, each vowel was replaced with another vowel that was not present in the word in the corresponding word sequence (e.g., “pain vend elle son” -> “peun vond olli san”).

Similar to Experiment 1, these three sets of 240 sequences (grammatical word, ungrammatical word, pseudoword) were divided into three subsets to create three lists of experimental stimuli presented to different participants. In this way, participants saw each sequence only once but were tested in all conditions with different sequences. Thus, for each participant, each set of four-word sequences occurred only once in its grammatically correct version (e.g., “elle vend son pain”), in the ungrammatical word version (e.g., “pain vend elle son”), or in the pseudoword sequence (e.g., “peun vond olli san”) (see Table 1).

#### Procedure

The procedure for stimulus presentation was identical to that used in Experiment 1.

#### Statistical power

With 90 participants and 80 items per condition the power of the experiment was largely superior to that recommended by Brysbaert and Stevens (2018).

### Results and discussion

First, the datasets from two participants were excluded, based on an overall error rate above 40%. We used generalized (logistic) LMEs to analyze our data, with items and participants as crossed random effects and including by-item and by-participant random intercepts and random slopes (Baayen et al., 2008; Barr et al., 2013). The logistic mixed-effects model included Grammaticality (grammatical word, ungrammatical word, vs. pseudoword) as fixed factor. The models were fitted with the glmer function from the lme4 package (Bates et al., 2014) in the R statistical computing environment (version 4.0.220, R Core Team, 2022). The maximal random effects structure that converged was one including by-participant and by-item random intercepts as well as by-participant random slopes for the Grammaticality factor. The following analyses were conducted taking the ungrammatical word condition as reference. We report regression coefficients (*b*), standard errors (*SE*s) and *z*-values for all factors. Fixed effects were deemed reliable if |*z*| > 1.96. Mean accuracy rates for each of the experimental conditions are presented in Table 3.

**Table 3:** Mean accuracy (% correct) in the grammatical word condition, the ungrammatical word condition, and the pseudoword condition in Experiment 2

Grammatical word	Ungrammatical word	Pseudoword
91.20 (5.91)	83.82 (7.69)	51.00 (10.44)

*Note*. Within-participant 95% confidence intervals are shown in parentheses

Accuracy was higher in the grammatical word condition than in the ungrammatical word condition (91.20% vs. 83.82%; *b* = 0.80, *SE* = 0.05, *z* = 14.08, *p* <.001), and accuracy was also higher in the ungrammatical word condition compared with the pseudoword condition (83.82% vs. 51.00%; *b* = − 1.89, *SE* = 0.04,* z* = − 42.26, *p* <.001).

In sum, we replicated the findings of Experiment 1 while replacing the nonword stimuli with pronounceable pseudoword stimuli. That is, we again observed an SSE on letter-in-string identification, with superior performance when the target letter was embedded in a word embedded in a grammatically correct word sequence. We also replicated the classic WSE – this time contrasting letter identification in words and pseudowords. When comparing the results of Experiment 2 with those of Experiment 1, it is clear that we also replicate the classic finding that the WSE is stronger when comparing words with nonwords (Experiment 1) than when comparing words with pseudowords (Experiment 2).

Given the evidence for a reduced WSE when comparing words versus pseudowords and words versus nonwords when tested in isolation in prior research (e.g., Adams, 1979; Grainger et al., 2003; McClelland, 1976; Rumelhart & McClelland, 1982) and also in a global context (Experiment 2), it is possible that the impact of sentence-level context (grammatical vs. ungrammatical) on the WSE might also be reduced when comparing words with nonwords versus words with pseudowords in the same experiment (see Table 1 for a summary of the conditions that were tested). Experiment 3 was designed to examine this possibility.

## Experiment 3

### Methods

#### Participants

A total of 64 participants (23 male) with a mean age of 21.6 years (SD = 4.45, min–max: 18–49 years) were recruited via the Prolific platform (Palan & Schitter, 2018). All participants were native speakers of French, with no history of neurological impairment, or developmental disorders. Prior to the beginning of the experiment, participants were informed that data would be collected anonymously, and they provided informed consent before the experiment was initiated.

#### Materials

The exact same materials used in the grammatically correct sentences and ungrammatical word sequences tested in Experiment 1 were used in Experiment 3. Here, target lexicality was manipulated. For each of the 240 grammatically correct sequences (e.g., elle vend son pain), we replaced the target word with either a pseudoword (e.g., “elle vond son pain”) or a nonword (e.g., “elle vbnd son pain”). Furthermore, we constructed ungrammatical sequences based on these three sets of 240 sequences by scrambling word order in the grammatical sequences but keeping the position of the word containing the target letter in the same position in the sequence (e.g., “pain vend elle son”; “pain vond elle son”; “pain vbnd elle son”) (see Table 1). These six sets of 240 sequences were separated into six subsets to create six lists of experimental stimuli presented to different participants. In this way, participants saw each sequence only once but were tested in all conditions with different sequences. Therefore, Grammaticality (grammatical vs. ungrammatical sequence) was crossed with Target Lexicality (word, pseudoword, nonword) in a 2 × 3 factorial design.

#### Procedure

The procedure for stimulus presentation was identical to that used in Experiment 1.

#### Statistical power

With 64 participants and 40 items per condition the power of the experiment was largely superior to that recommended by Brysbaert and Stevens (2018).

### Results and discussion

First, the datasets from eight participants were excluded, based on an overall error rate above 40%. One more participant was excluded because he/she did not complete the experiment. We used generalized (logistic) LMEs to analyze our data, with items and participants as crossed random effects and including by-item and by-participant random intercepts and random slopes (Baayen et al., 2008; Barr et al., 2013). The logistic mixed-effects model included Grammaticality (grammatical sequence vs. ungrammatical sequence) and Target Lexicality (word, pseudoword, nonword) as fixed factors. The models were fitted with the glmer function from the lme4 package (Bates et al., 2014) in the R statistical computing environment (version 4.0.220, R Core Team, 2022). The maximal random effects structure that converged was one including by-participant and by-item random intercepts as well as by-participant random slopes for the Grammaticality × Lexicality interaction. The following analyses were conducted taking the ungrammatical condition as reference for the Grammaticality factor and the word condition as reference for the Target Lexicality factor. We report regression coefficients (*b*), standard errors (*SE*s) and *z*-values for all factors. Fixed effects were deemed reliable if |*z*| > 1.96. Mean accuracy rates for each of the experimental conditions are presented in Table 4.

**Table 4:** Mean accuracy (% correct) in the grammatical and in the ungrammatical conditions as a function of Target Lexicality in Experiment 3

	Grammatical	Ungrammatical
Word	83.45 (9.82)	77.09 (11.10)
Pseudoword	75.04 (11.43)	68.63 (12.26)
Nonword	59.04 (12.99)	53.50 (13.18)

*Note*. Within-participant 95% confidence intervals are shown in parentheses

Accuracy was higher in the grammatical condition than in the ungrammatical condition (72.52% vs. 66.41%; *b* = 0.60, *SE* = 0.10, *z* = 5.96, *p* <.001). The effect of Lexicality was also significant, with a higher accuracy rate for word targets than for pseudoword targets (80.27% vs. 71.83%; *χ*^*2*^(1) = 78.03, *p* <.001), and a higher accuracy rate for word targets than for nonword targets (80.27% vs. 56.27%; *χ*^*2*^(1) = 231.17, *p* <.001). Accuracy was also significantly higher for pseudoword targets than for nonword targets (71.83% vs. 56.27%; *χ*^*2*^(1) = 136.27, *p* <.001). The only interaction that reached significance was the one including Grammaticality (grammatical vs. ungrammatical) × Lexicality (word vs. nonword) (*b* = − 0.34, *SE* = 0.12, *z* = − 2.55, *p* =.010). This interaction reflects the fact that the size of the SSE was greater for letters-in-words (*χ*^*2*^(1) = 35.00, *p* <.001) than for letters-in-nonwords (*χ*^*2*^(1) = 15.77, *p* <.001).

In Experiment 4 we provide a test of the same set of word, pseudoword, and nonword stimuli as tested in Experiment 3, but this time presented in isolation (i.e., in the absence of a surrounding context). We aimed to replicate the classic pattern of word and pseudoword superiority effects, while providing a baseline with which the effects of global context (grammatical/ungrammatical sequences) can be further evaluated. This Experiment is therefore essential for the cross-experiment analyses to be performed in a following section.

## Experiment 4

### Methods

#### Participants

A total of 90 participants (49 male) with a mean age of 34 years (SD = 10.75, min–max: 20–65 years) were recruited via the Prolific platform (Palan & Schitter, 2018). All participants were native speakers of French, with no history of neurological impairment or developmental disorders. Prior to the beginning of the experiment, participants were informed that data would be collected anonymously, and they provided informed consent before the experiment was initiated.

#### Materials

The exact same set of word, pseudoword, and nonword stimuli (N = 720) as tested in Experiment 3 were used in Experiment 4. Here stimuli were presented in isolation resulting in a single factor (Lexicality: word, pseudoword, nonword) with 240 stimuli per condition. These three sets of 240 stimuli were divided into three subsets to create three lists of experimental stimuli presented to different participants. In this way, participants saw each stimulus in a given triplet only once (i.e., tested for target “v” in either “over,” “avir,” or “pvgr”), but were tested in all conditions with different stimuli.

#### Procedure

The procedure for stimulus presentation was identical to that used in Experiment 1, except that there was no surrounding grammatical or ungrammatical context.

#### Statistical power

With 90 participants and 240 items per condition, the power of the experiment was largely superior to that recommended by Brysbaert and Stevens (2018).

### Results and discussion

First, the datasets from two participants were excluded, based on an overall error rate above 40%. We used generalized (logistic) LMEs to analyze our data, with items and participants as crossed random effects and including by-item and by-participant random intercepts and random slopes (Baayen et al., 2008; Barr et al., 2013). The logistic mixed-effects model included Target Lexicality (word, pseudoword, nonword) as fixed-factor. The models were fitted with the glmer function from the lme4 package (Bates et al., 2014) in the R statistical computing environment (version 4.0.220, R Core Team, 2022). The maximal random effects structure that converged was one including by-participant and by-item random intercepts as well as by-participant random slopes for the Lexicality factor. The following analyses were conducted taking the word condition as reference for the Target Lexicality factor. We report regression coefficients (*b*), standard errors (*SE*s) and *z*-values for all factors. Fixed effects were deemed reliable if |*z*| > 1.96. Mean accuracy rates for each of the experimental conditions are presented in Table 5.

**Table 5:** Mean accuracy (% correct) for each condition of Target Lexicality in Experiment 4

Word	Pseudoword	Nonword
93.62 (5.10)	83.50 (7.75)	52.78 (10.43)

*Note*. Within-participant 95% confidence intervals are shown in parentheses

Letter identification accuracy was higher when the target letter was presented in the context of a word than in the context of a pseudoword (93.62% vs. 83.50%; *χ*^*2*^(1) = 189.01, *p* <.001). Similarly, accuracy was higher when the target letter was presented in the context of a word than when presented in the context of a nonword (93.62% vs. 52.78%; *χ*^*2*^(1) = 718.80, *p* <.001). Accuracy was significantly higher in the context of a pseudoword than in the context of a nonword (83.50% vs. 52.78%; *χ*^*2*^(1) = 710.02, *p* <.001).

Experiment 4 therefore replicated the classic pattern of word and pseudoword superiority effects reported in prior studies of the WSE. As typically observed in prior studies using isolated stimuli (e.g., Adams, 1979; Grainger et al., 2003; McClelland, 1976; Rumelhart & McClelland, 1982), the WSE is about twice as large when words are compared with nonwords relative to the word-pseudoword contrast.

#### Cross-experiment analyses

Here we combine the results of Experiment 4 with those of Experiment 3 in order to provide a more complete view on how word and pseudoword superiority effects are impacted by the nature of the global context surrounding the embedding stimulus. We introduce a terminology that distinguishes between the *global context* (grammatical vs. ungrammatical) and the *local context* (word, pseudoword, nonword). By adding the results of Experiment 4 to those of Experiment 3 we can examine how the contrast between words versus pseudowords versus nonwords (i.e., the local context) varies a function of the nature of the global context surrounding the embedding stimulus, including a no-context condition. Note that the same set of embedding words, pseudowords, and nonwords were used in these two experiments.

**Fig. 2:**
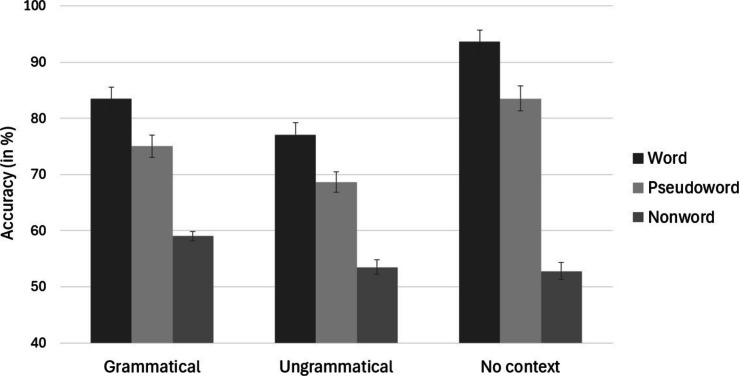
Comparison of effects of local context (word, pseudoword, nonword) on letter identification accuracy as a function of the global context (grammatical, ungrammatical, no-context). The data for the grammatical and ungrammatical conditions are from Experiment 3, and those for the no-context condition from Experiment 4. Error bars are 95% confidence intervals

Combining all six conditions tested in Experiment 3 with the three conditions tested in Experiment 4 led to a 3 (Local context: word vs. pseudoword vs. nonword) × 3 (Global context: grammatical, ungrammatical, no context) factorial design. We used generalized (logistic) LMEs, with items and participants as crossed random effects and including by-item and by-participant random intercepts and random slopes (Baayen et al., 2008; Barr et al., 2013). The logistic mixed-effects model included Global context (grammatical sequence, ungrammatical sequence, no context) and Local context (word, pseudoword, nonword) as fixed factors. The models were fitted with the glmer function from the lme4 package (Bates et al., 2014) in the R statistical computing environment (version 4.0.220, R Core Team, 2022). The maximal random effects structure that converged was one including by-participant and by-item random intercepts as well as by-participant random slopes for the Local context factor and by-item random slopes for the Global context factor. The following analyses were conducted taking the ungrammatical condition as reference for the Global context factor and the word condition as reference for the Local context factor. Figure 2 illustrates how effects of local context (word, pseudoword, nonword) on letter identification vary as a function of the global context that surrounds the embedding stimulus.

There was a main effect of Global context (*χ*^*2*^(2) = 87.03, *p* <.001) as well as a main effect of Local context (*χ*^*2*^(2) = 134.98, *p* <.001). Crucially, the Global context × Local context interaction was significant, *χ*^*2*^(4) = 148, *p* <.001. Upon examination of Fig. 2, it appears that there are two factors driving the interaction effect. First, there is the sentence superiority effect already reported in Experiment 3 (superior letter identification in grammatical contexts vs. ungrammatical contexts). Then there is the overall effect of the presence or not of a global context. The latter effect is most clearly illustrated by the contrast between the ungrammatical context of Experiment 3 and the no-context condition tested in Experiment 4 (see Fig. 2). A follow-up analysis of the results of the ungrammatical condition in Experiment 3 and the no-context condition of Experiment 4 revealed a significant interaction between Local context (i.e., word, pseudoword, nonword) and Global context (i.e., ungrammatical context vs. no-context), *χ*^*2*^(2) = 150.75, *p* <.001. While accuracy of letter identification was greater in the no-context than in the ungrammatical context for words (93.62% vs. 77.09%, respectively, *χ*^*2*^(1) = 56.79, *p* <.001) and pseudowords (83.50% vs. 68.63%, respectively, *χ*^*2*^(1) = 29.86, *p* <.001), this was not the case for nonword stimuli (52.78% vs. 53.50%, respectively, *χ*^*2*^(1) < 0.1, *p* >.1). We discuss the implications of the results of these cross-experiment analyses in the General discussion.

## General discussion

The primary result of the present study is the finding of a novel sentence superiority effect (SSE) on letter-in-word identification. Letter identification accuracy in words (e.g., the letter V in OVER) was greater when the embedding word (OVER) was itself embedded in a grammatically correct sequence of words (HE LOOKS OVER THERE) compared with an ungrammatical sequence of words (THERE HE OVER LOOKS). This effect was found consistently in Experiments 1, 2, and 3. This is an important result since it demonstrates that even when participants are performing a task that uniquely requires single-letter identification, the global structure of the sequence (grammatical vs. ungrammatical) impacts on participants’ responses to single letters. That is, although the global context is irrelevant for the task, it determines the manner in which the local context (word, pseudoword, nonword) impacts on single letter identification.

We would argue that this is strong evidence that participants cannot inhibit a natural tendency to perform parallel word processing when presented with sequences of words. That is, when presented with the sequence “HE LOOKS OVER THERE” and post-cued to identify the letter “V,” participants responses are not only influenced by the local context (i.e., the target letter is part of the word “over”), but also the global context (i.e., the word “over” is part of a grammatically correct sequence of words). Within the theoretical framework of the cascaded interactive-activation model of reading described in the *Introduction*, this could arise by word-level processing providing feedback to on-going letter-level processing (e.g., activation of the word “over” would provide support that the second letter in that sequence of letters is the letter “v”), and by sentence-level processing providing feedback to word-level processing (i.e., the grammaticality of the global context “he looks over there” would provide support for identification of the word “over” in that sequence). It is the combination of these two influences that can account for the present findings.

Crucially, the present results provide further support for the notion of parallel processing not only at the letter-in-word level (as implemented in McClelland & Rumelhart’s interactive-activation model), but also at the word-in-sentence level (see Snell & Grainger, 2019, for a summary of prior evidence for parallel word processing). Such evidence for parallel word processing (within the limits of visual acuity, spatial attention, and crowding) flies in the face of strictly serial one-word-at-time accounts of skilled reading (e.g., Reichle et al., 1998). Furthermore, related research with young readers (Brossette et al., 2025) suggests that multi-word processing skills strongly depend on first achieving fluency in single-word processing, and that this is attained by around grade 3 in primary education in France. This timing corresponds with the evidence obtained in on-going work in our group (Massol et al., in prep.), that the WSE emerges in grade 3 children. Therefore, the ability to process both letters and words (to a certain extent) in parallel would emerge around the same time during reading development and likely constitute a key factor in the emergence of skilled reading. This, however, remains to be tested in appropriate developmental studies examining the emergence of the WSE and the SSE in the same group of children.

We also replicated the standard finding that the WSE is greater when contrasting letter-in-word versus letter-in-nonword identification compared with a word versus pseudoword contrast (e.g., Adams, 1979; Grainger et al., 2003; McClelland, 1976; Rumelhart & McClelland, 1982; see also Carr, 1986). This pattern was predicted by McClelland and Rumelhart’s (1981) interactive-activation model in that pseudoword stimuli can partially activate real words more than can nonword stimuli, and this partial activation provides feedback to on-going letter-identification processes. Alternative bottom-up explanations are possible (see, e.g., Grainger & Jacobs, 1994), but here we prefer the more parsimonious explanation provided by McClelland and Rumelhart since this also provided a natural explanation of the SSE with the same mechanism operating between the word and sentence level as between the letter and word level (see Brossette et al., 2022, and Grainger, 2024, for a discussion of the parallels between letter-word and word-sentence processing).

The key question raised in the present study concerns how the effects of local context (i.e., word, pseudoword, nonword) combine with the effects of global context (i.e., grammatical, ungrammatical) in determining the ease with which participants can identify a single letter within a sequence of four spatially distinct letter strings. In other words, can SSEs combine with WSEs to increase the identifiability of target letters? As argued above, our results suggest that there is indeed an additive influence, with the effects of global context augmenting effects driven by local context. However, as can be seen in Fig. 2, there is another factor at play. This is the overall interfering effect of a global context on letter-in-word and letter-in-pseudoword identification. This interfering effect was seen to have an impact on letter-in-word and letter-in pseudoword identification but not letter-in-nonword identification. Such an interfering effect of global context has already been found in studies using the reading version of the flankers task (see Grainger, 2024, for a summary). That is, when asked to classify a central target as being a word or not, the presence of flanking stimuli to the left and to the right of the central target interferes in participants’ decisions made to central targets. Therefore, effects of flanker relatedness reflect a compensation for the overall effect of flanker interference (e.g., Cauchi et al., 2020; Snell & Grainger, 2018). In line with flanker studies, the SSE seen in letter-in-string identification in the present work reflects how the nature of the global context (grammatical vs. ungrammatical) mitigates the overall interfering influence of global context on letter-in-word and letter-in-pseudoword processing.

## Conclusions

In four post-cued partial report letter-identification experiments we replicated the classic pattern of word superiority effects – that is, letter-identification accuracy is better in words compared with pseudowords, and better in pseudowords compared with nonwords. Crucially, we also demonstrated that the global context surrounding the letter string in which the target letter was embedded also impacted letter-identification accuracy. Letter-in-word identification was facilitated when the word was itself part of a grammatically correct sentence compared with an ungrammatical sequence of words. We propose that this novel sentence superiority effect seen in single letter-identification accuracy reflects the operation of cascaded, interactive processing between letter, word, and sentence level representations.

## Data Availability

The authors confirm the availability of shared data. The datasets are accessible on the Open Science Framework website (https://osf.io/ke29n/).

## Footnotes

1 The only notable difference in results obtained with a 2 AFC procedure and those obtained with a simple post-cued partial report procedure in Tydgat and Grainger (2009) concerned performance at the final position, where 2 AFC tended to reduce the final-letter superiority (relative to inner letters) that is typically found with post-cued partial report. Note that the present study was not aimed at studying serial position effects and never tested end-letters.

## Code availability

Codes for data analyses can be found via the Open Science Framework (https://osf.io/ke29n/).
